# The mediating role of health literacy in the relationship between social support and glycemic control among rural patients with diabetes in Western China: a cross-sectional SEM study

**DOI:** 10.3389/fendo.2026.1733826

**Published:** 2026-05-20

**Authors:** Yelan Huang, Guifen Fu, Yanping Zhang, Jingfeng Chen, Chaoqun Bai, Xiaomin Xian

**Affiliations:** 1 Faculty of Nursing, Guangxi University of Chinese Medicine, Nanning, China; 2 Department of Nursing, Guangxi Academy of Medical Sciences and the People’s Hospital of Guangxi Zhuang Autonomous Region, Nanning, China; 3 Department of Geriatric Endocrinology and Metabolism, Guangxi Academy of Medical Sciences and the People’s Hospital of Guangxi Zhuang Autonomous Region, Nanning, China; 4 Nursing Department, Faculty of Chinese Medicine Science, Guangxi University of Chinese Medicine, Nanning, China

**Keywords:** diabetes, HbA1c level, health literacy, rural population, social support, structural equation modeling

## Abstract

**Background:**

Social support and health literacy are important factors associated with glycemic control in patients with diabetes, but the mediating role of health literacy in rural populations remains unclear.

**Methods:**

We conducted a cross-sectional survey of 2,280 rural patients with diabetes across five regions in Guangxi using the Social Support Scale and Health Literacy Scale. Data were analyzed using Spearman correlation and structural equation modeling (SEM). A covariate-adjusted SEM was further estimated by including age, sex, education level, household income, disease duration, number of complications, BMI, and treatment type.

**Results:**

Among 2,178 valid questionnaires, the mean social support score was 26.70 ± 9.93, the mean health literacy score was 30.47 ± 9.36, and the mean HbA1c level was 8.60 ± 2.18%. Only 22.64% of participants had HbA1c levels below 7.0%, indicating generally poor glycemic control. Spearman correlation analysis showed that both social support and health literacy were significantly negatively correlated with HbA1c level (*p* < 0.01). The covariate-adjusted SEM showed acceptable to good model fit (CFI = 0.975, TLI = 0.940, RMSEA = 0.043, and SRMR = 0.018). In the adjusted model, social support remained positively associated with health literacy (β = 0.471, *p* < 0.001), and both social support (β= -0.308, *p* < 0.001) and health literacy (β= -0.353, *p* < 0.001) were negatively associated with HbA1c level. Bootstrap analysis indicated a significant indirect effect of social support on HbA1c through health literacy (estimate = -0.037, 95% CI: -0.042 to -0.031), accounting for 35.05% of the total effect.

**Conclusion:**

Health literacy plays a significant mediating role in the relationship between social support and HbA1c level among rural patients with diabetes in western China. Strengthening social support and improving health literacy may help improve glycemic control in rural diabetes management.

## Introduction

Diabetes mellitus is one of the most serious public health challenges worldwide and is characterized by hyperglycemia caused by insufficient insulin secretion, impaired insulin action, or both. Poor HbA1c control markedly increases the risk of cardiovascular, renal, and other systemic complications, placing substantial burdens on patients, families, and health systems (1). According to the 11th edition of the IDF Diabetes Atlas, the global prevalence of diabetes among adults aged 20–79 years is 10.5%, and the number of people living with diabetes is projected to reach 783 million by 2045 (2). China has the largest number of people with diabetes worldwide, and the prevalence among adults remains high (3). In rural areas, the burden of diabetes is further aggravated by changing lifestyles, social isolation, low literacy, economic constraints, and limited healthcare accessibility (4–6). These challenges highlight the need to identify modifiable psychosocial and behavioral factors associated with glycemic control among rural patients with diabetes.

Social support and health literacy are two important factors that may influence HbA1c control in patients with diabetes (7). Social support refers to an individual’s perception and actual receipt of emotional, informational, and practical resources from social networks, including family members, friends, neighbors, and healthcare providers. Adequate social support may promote healthy behaviors, improve treatment adherence, enhance psychological well-being, and reduce adverse outcomes during chronic disease management (8, 9). However, previous studies have suggested that Chinese patients with diabetes often receive insufficient social support, particularly in resource-limited settings (10–12). Health literacy refers to an individual’s ability to obtain, understand, evaluate, and apply health information and services to make appropriate health decisions. Limited health literacy has been associated with poorer treatment adherence, weaker self-management, higher HbA1c levels, and greater complication risks (13–15). Therefore, both social support and health literacy are likely to play important roles in diabetes management, especially in populations with limited healthcare and educational resources.

Theoretically, social support may influence glycemic control through both direct and indirect pathways. Directly, supportive social relationships may encourage healthier behaviors, facilitate treatment engagement, and improve psychological coping. Indirectly, social support may enhance patients’ ability to access, interpret, and use health information, thereby improving health literacy. Improved health literacy may in turn strengthen diabetes self-management, including dietary regulation, medication adherence, condition monitoring, and communication with healthcare professionals, ultimately contributing to better HbA1c control (16–20). This framework suggests that health literacy may serve as an important mediator linking social support to glycemic outcomes.

Although previous studies have examined the associations among social support, health literacy, self-management, and diabetes-related outcomes, the mediating role of health literacy has not been adequately clarified in rural populations with diabetes (16, 17, 19, 21). For example, a recent path analysis among rural individuals with type 2 diabetes in eastern China showed that social support directly and indirectly influenced self-management and quality of life through self-efficacy (22). Another path analysis reported that both diabetes health literacy and social support were associated with self-care behaviors and quality of life in patients with type 2 diabetes (22). In addition, recent work in Xi’an found that social support was associated with medication adherence and glycemic control (14), while a mixed-methods study from Quito highlighted the importance of social support for diabetes management in low-resource settings (11, 12). Together, these findings support the relevance of examining both the direct and indirect roles of social support in diabetes care.

Compared with populations in more urbanized or better-resourced settings, rural patients may face poorer healthcare accessibility, lower educational attainment, fewer opportunities for health information exchange, and weaker social support networks, all of which may affect both health literacy and glycemic control (23–25). Recent studies have also identified barriers in primary care diabetes management (26) and difficulties in complication-risk perception among rural patients (27), suggesting that rural health systems face challenges beyond individual-level behavior alone. Therefore, findings from urban or mixed-population studies may not be fully generalizable to rural populations in Guangxi.

Against this background, the present study focused on rural patients with diabetes in Guangxi, where socioeconomic disadvantage, geographical barriers, and disparities in healthcare resources may jointly affect social support, health literacy, and HbA1c control. Guangxi is a multi-ethnic, economically less-developed, and geographically diverse region, which may further shape patients’ access to health information, patterns of social support, and diabetes management behaviors. Structural equation modeling was used because it allows the simultaneous estimation of direct and indirect pathways and is therefore well suited to testing the hypothesized mediating role of health literacy in the relationship between social support and HbA1c level. The aim of this study was to examine the mediating role of health literacy in the relationship between social support and glycemic control (HbA1c level) among rural patients with diabetes in western China, and to provide evidence for targeted interventions in rural diabetes management.

## Materials and methods

### Study population

This cross-sectional study was conducted between January 2022 and July 2022. Using a multi-stage stratified sampling method, we recruited participants from five regions of Guangxi. In the first stage, five cities representing different geographic areas of Guangxi were randomly selected: Nanning (central), Guilin (northern), Hechi (western), Chongzuo (southern), and Yulin (eastern). In the second stage, one endocrinology department was randomly selected from each of three counties within each city, yielding a total of 15 departments. The target sample size was 152 participants per department, resulting in a planned sample of 2,280 patients. Eligible participants were adults aged ≥18 years who met the 2022 Chinese Diabetes Association diagnostic criteria for diabetes and were local residents, defined as having both household registration and current residence within the study area. Exclusion criteria included gestational diabetes, cognitive impairment or diagnosed mental illness, and severe diabetes complications or acute-phase conditions (28). Within each participating hospital, eligible patients were recruited by convenience sampling during the study period. Investigators collaborated with hospital staff, who first approached potential participants. Patients who met the eligibility criteria and agreed to participate were enrolled after providing written informed consent. Institutional consent was obtained from all participating hospitals.

### Survey instruments

#### General information questionnaire

The researcher-developed questionnaire contained two main sections: demographic information (age, sex, marital status, educational attainment, per capita household income, body weight, and BMI) and disease-related information (duration of diabetes, HbA1c level, number of complications, use of oral hypoglycemic agents, and insulin therapy).

#### Health literacy scale

The Health Literacy Scale (HLS), developed by Ishikawa (29), was designed to assess health literacy among patients with diabetes. Zhao Xiaoyan (30) translated and culturally adapted the scale into Chinese in 2021 and confirmed its reliability and validity. The HLS includes three dimensions—functional, communicative, and critical health literacy—and comprises 14 items. Each item is rated on a 4-point Likert scale from 1 (not at all) to 4 (most of the time). The functional health literacy dimension is reverse-scored, yielding a total score ranging from 14 to 56, with higher scores indicating better health literacy. In the present study, the Chinese version showed good internal consistency (Cronbach’s α = 0.868).

#### Social support rating scale

The Social Support Rating Scale (SSRS), developed by Xiao Shuiyuan (31), assesses social support across three dimensions: subjective support, objective support, and support utilization. The scale contains 10 items. Items 1–4 and 8–10 are single-choice questions scored from 1 to 4. Item 5 consists of five sub-items, each scored from 1 to 4, with the sub-item scores summed. For items 6 and 7, 0 points indicate no support sources, and each available source adds 1 point. The total score is the sum of all 10 items, with higher scores indicating greater social support. Scores are categorized as low (≤22), moderate (23–44), and high (45–66). In this study, the SSRS demonstrated good reliability (Cronbach’s α = 0.838).

### Survey methods

Investigators from 15 hospitals completed standardized survey administration training before data collection. The investigators recorded general patient information and HbA1c levels, administered questionnaires, compiled data into Excel spreadsheets and then subsequently transmitted to the researcher via the internet. Two researchers independently validated and processed the data.

### Data analysis

Data were analyzed using SPSS 26.0 and AMOS 24.0. Frequencies and percentages were used to describe the demographic and disease-related characteristics of rural patients with diabetes, whereas means ± standard deviations were used to summarize social support, health literacy, and HbA1c level. Spearman correlation analysis was performed to examine the relationships among social support, health literacy, and HbA1c level. Additional Spearman correlation analyses were conducted to assess the associations between the subscales of social support, health literacy, and HbA1c level.

Structural equation modeling was used to test the mediating role of health literacy in the relationship between social support and HbA1c level. Mediation effects were estimated using the bootstrap method with 5,000 resamples and 95% confidence intervals. A covariate-adjusted structural equation model was further estimated, with age, sex, education level, household income, disease duration, number of complications, BMI, and treatment type included as exogenous covariates predicting both health literacy and HbA1c level. Treatment type was reconstructed from oral hypoglycemic agent use and insulin therapy and entered as dummy variables. Model fit was evaluated using χ²/df, CFI, TLI, RMSEA, and SRMR.

## Results

### Sociodemographic characteristics of the participants

The demographic and clinical characteristics of the participants are presented in **Table 1**. A total of 2,178 rural patients with diabetes completed the survey, yielding a response rate of 95.53%. Of these, 1,204 (55.28%) were male and 974 (44.72%) were female, with a mean age of 63.25 ± 12.71 years. Most participants were married (n = 2,019, 92.70%), with one missing value for marital status. The mean duration of diabetes was 7.96 ± 4.07 years. The mean body weight was 59.22 ± 10.12 kg, and the mean BMI was 23.06 ± 3.16 kg/m². In terms of household income, 1,356 participants (62.26%) were in the low-income group and 822 (37.74%) were in the high-income group. Regarding treatment type, 521 participants (23.92%) received no medication, 744 (34.16%) received oral medication only, 308 (14.14%) received insulin only, and 605 (27.78%) received both oral medication and insulin. The mean HbA1c level was 8.60 ± 2.18%. For a more detailed description of glycemic control, HbA1c levels were categorized into four groups: <7.0% (n = 493, 22.64%), 7.0–7.9% (n = 379, 17.40%), 8.0–8.9% (n = 402, 18.46%), and ≥9.0% (n = 904, 41.51%). This distribution indicated substantial heterogeneity in glycemic control within the study population.

**Table 1:** Demographic and clinical characteristics of 2,178 rural patients with diabetes.

Variable	Category	n	%/mean ± SD
Age (years)			63.25 ± 12.71
	18–44	158	7.25
	45–59	672	30.85
	≥60	1348	61.89
Sex	Male	1204	55.28
	Female	974	44.72
Marital status	Unmarried/widowed	158	7.25
	Married	2019	92.70
	Missing	1	0.05
Education level	Illiterate/primary school	760	34.89
	Junior high school	684	31.40
	Senior high school/college	687	31.54
	Bachelor’s degree or above	47	2.16
Per capita household income	Low	1356	62.26
	High	822	37.74
Disease duration (years)			7.96 ± 4.07
	<5 years	465	21.35
	5–10 years	1134	52.07
	>10 years	579	26.58
Body weight (kg)			59.22 ± 10.12
BMI (kg/m²)			23.06 ± 3.16
BMI category	<18.5	126	5.79
	18.5–24.9	1545	70.94
	25.0–29.9	441	20.25
	≥30.0	66	3.03
Number of complications	None	607	27.87
	1	895	41.09
	2	554	25.44
	3	112	5.14
	4	10	0.46
Treatment type	No medication	521	23.92
	Oral medication only	744	34.16
	Insulin only	308	14.14
	Oral medication + insulin	605	27.78
HbA1c level (%)			8.60 ± 2.18
	<7.0	493	22.64
	7.0–7.9	379	17.40
	8.0–8.9	402	18.46
	≥9.0	904	41.51

BMI, body mass index; HbA1c, glycated hemoglobin. Treatment type was reconstructed from oral hypoglycemic agent use and insulin therapy into four categories: no medication, oral medication only, insulin only, and oral medication + insulin. One value was missing for marital status.

### Correlations of the study variable

The correlations among the study variables are presented in **Table 2**. The mean social support score was 26.70 ± 9.93. The median scores of its three dimensions were 8 (IQR: 7–13) for subjective support, 7 (IQR: 5–12) for objective support, and 7 (IQR: 6–8) for support utilization. The mean health literacy score was 30.47 ± 9.36, including functional health literacy (11.17 ± 3.76), communicative health literacy (10.64 ± 3.92), and critical health literacy (8.65 ± 3.07). Social support was positively correlated with health literacy (r = 0.472, *p* < 0.01). In contrast, both social support (r = -0.447, *p* < 0.01) and health literacy (r = -0.617, *p* < 0.01) were negatively correlated with HbA1c level.

**Table 2:** Correlation analysis of social support, health literacy, and HbA1c level.

Item	Result	Social support	Health literacy	HbA1c level
Social support	26.70 ± 9.93 (points)	1		
Health literacy	30.47 ± 9.36 (points)	0.472^*^	1	
HbA1c level	8.60 ± 2.18 (%)	-0.447^*^	-0.617^*^	1

*p < 0.01 (two-tailed).

**Figure 1:**
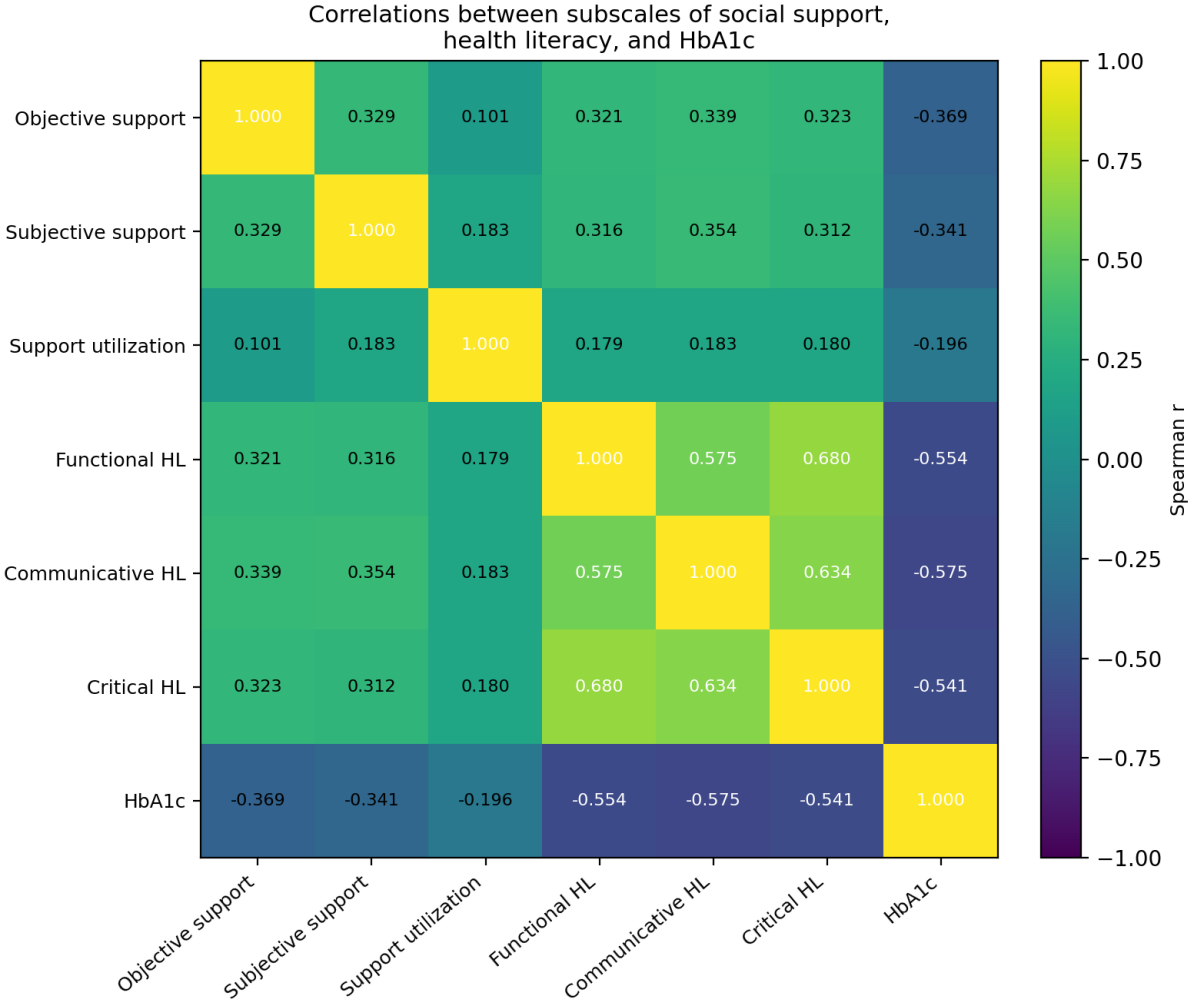
Heatmap of Spearman correlations among social support subscales, health literacy subscales, and HbA1c level.

To further examine these relationships, Spearman correlation analyses were performed for the subscales of social support and health literacy (**Table 3** and **Figure 1**). Among the social support subscales, objective support (r = -0.369, *p* < 0.01), subjective support (r = -0.341, *p* < 0.01), and support utilization (r = -0.196, *p* < 0.01) were all negatively correlated with HbA1c level. Similarly, all three health literacy subscales were significantly negatively correlated with HbA1c level, including functional health literacy (r = -0.554, *p* < 0.01), communicative health literacy (r = -0.575, *p* < 0.01), and critical health literacy (r = -0.541, *p* < 0.01). Overall, the health literacy subscales showed stronger correlations with HbA1c level than the social support subscales.

**Table 3:** Correlations between subscales of social support, health literacy, and HbA1c.

Variable	Objective support	Subjective support	Support utilization	Functional HL	Communicative HL	Critical HL	HbA1c level
Objective support	1.000	0.329**	0.101**	0.321**	0.339**	0.323**	-0.369**
Subjective support	0.329**	1.000	0.183**	0.316**	0.354**	0.312**	-0.341**
Support utilization	0.101**	0.183**	1.000	0.179**	0.183**	0.180**	-0.196**
Functional HL	0.321**	0.316**	0.179**	1.000	0.575**	0.680**	-0.554**
Communicative HL	0.339**	0.354**	0.183**	0.575**	1.000	0.634**	-0.575**
Critical HL	0.323**	0.312**	0.180**	0.680**	0.634**	1.000	-0.541**
HbA1c level	-0.369**	-0.341**	-0.196**	-0.554**	-0.575**	-0.541**	1.000

HL, health literacy. **p < 0.01(two-tailed).HbA1c was analyzed as a continuous variable.

### Analysis of the mediating effect of health literacy between social support and HbA1c control

To account for potential confounding, a covariate-adjusted structural equation model was estimated. Age, sex, education level, household income, disease duration, number of complications, BMI, and treatment type were included as exogenous covariates predicting both health literacy and HbA1c level. The adjusted model showed acceptable to good fit (χ² = 224.746, df = 44, χ²/df = 5.108, CFI = 0.975, TLI = 0.940, RMSEA = 0.043, and SRMR = 0.018) (**Table 4**).

**Table 4:** Fit indices for the covariate-adjusted SEM.

Model	χ²	df	χ²/df	CFI	TLI	RMSEA	SRMR	Model fit
Covariate-adjusted latent SEM	224.746	44	5.108	0.975	0.940	0.043	0.018	Acceptable

CFI, comparative fit index; TLI, Tucker-Lewis index; RMSEA, root mean square error of approximation; SRMR, standardized root mean square residual. The latent SEM was specified using the three social support subscales and the three health literacy subscales as indicators.

**Figure 2:**
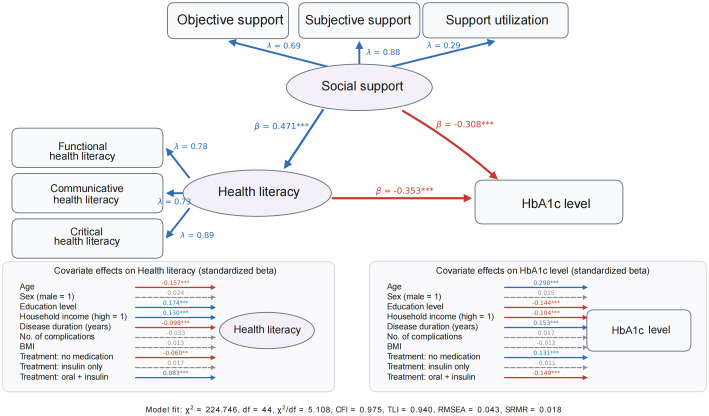
Covariate-adjusted structural equation model of social support, health literacy, and HbA1c level. **p < 0.01, ***p < 0.001; dashed gray arrows indicate non-significant paths. Treatment reference group=oral medication only. Values on measurement arrows are standardized loadings (λ); values on structural and covariate arrows are standardized coefficients (β).

As shown in **Table 5** and **Figure 2**, social support remained positively associated with health literacy after adjustment for covariates (B = 0.444, β = 0.471, *p* < 0.001). In the HbA1c equation, both social support (B = -0.068, β = -0.308, *p* < 0.001) and health literacy (B = -0.082, β = -0.353, *p* < 0.001) remained negatively associated with HbA1c level. Among the covariates, age, education level, household income, disease duration, and treatment type were significantly associated with HbA1c level, whereas sex, number of complications, and BMI were not. For health literacy, age, education level, household income, disease duration, and treatment type were significant predictors, whereas sex, number of complications, and BMI were not.

**Table 5:** Standardized Structural Paths in the Covariate-Adjusted Model.

Predictor	Health literacy B (SE)	Health literacy β	Health literacy p	HbA1c B (SE)	HbA1c β	HbA1c p
Social support	0.444 (0.018)	0.471	<0.001	-0.068 (0.004)	-0.308	<0.001
Health literacy	—	—	—	-0.082 (0.004)	-0.353	<0.001
Age	-0.116 (0.014)	-0.157	<0.001	0.051 (0.003)	0.298	<0.001
Sex (male = 1)	0.443 (0.336)	0.024	0.187	0.068 (0.061)	0.015	0.270
Education level	1.344 (0.139)	0.174	<0.001	-0.261 (0.026)	-0.144	<0.001
Household income (high = 1)	2.513 (0.347)	0.130	<0.001	-0.468 (0.064)	-0.104	<0.001
Disease duration (years)	-0.224 (0.041)	-0.098	<0.001	0.082 (0.008)	0.153	<0.001
No. of complications	-0.346 (0.191)	-0.033	0.070	0.042 (0.035)	0.017	0.232
BMI	0.040 (0.052)	0.013	0.450	-0.009 (0.010)	-0.012	0.371
Treatment: no medication	-1.311 (0.443)	-0.060	0.003	0.668 (0.081)	0.131	<0.001
Treatment: insulin only	0.463 (0.523)	0.017	0.376	-0.067 (0.096)	-0.011	0.482
Treatment: oral + insulin	1.737 (0.427)	0.083	<0.001	-0.727 (0.078)	-0.149	<0.001

Treatment type was reconstructed from oral hypoglycemic agent use and insulin therapy into four categories: no medication, oral medication only, insulin only, and oral medication plus insulin, with oral medication only as the reference group. B indicates the unstandardized regression coefficient, SE the standard error, and β the standardized regression coefficient. Standardized coefficients were used to compare the relative strength of associations across predictors. p values indicate statistical significance.

Bootstrap analysis based on 5,000 resamples showed that the indirect effect of social support on HbA1c through health literacy remained significant after covariate adjustment (estimate = -0.037, 95% CI: -0.042 to -0.031), indicating partial mediation (**Table 6**). The direct effect of social support on HbA1c was -0.068 (95% CI: -0.076 to -0.060), and the total effect was -0.104 (95% CI: -0.111 to -0.097). The indirect effect accounted for 35.05% of the total effect.

**Table 6:** Direct, indirect, and total effects of social support on HbA1c level.

Effect	Estimate	Boot 95% CI lower	Boot 95% CI upper	Proportion mediated
Indirect effect (social support → health literacy → HbA1c)	-0.037	-0.042	-0.031	35.05%
Direct effect (social support → HbA1c)	-0.068	-0.076	-0.060	—
Total effect	-0.104	-0.111	-0.097	—

Bias-corrected percentile confidence intervals were derived from 5,000 bootstrap resamples. The indirect effect is statistically significant because the 95% confidence interval does not include 0.

## Discussion

### Current status of social support, health literacy and glycemic control among rural diabetic patients in Guangxi

In this study, the mean total social support score among rural patients with diabetes in Guangxi was 26.70 (SD = 9.93), indicating a moderate level of social support, which was consistent with the findings reported by Sun et al. (21). Scores for subjective support, objective support, and support utilization were all relatively low, suggesting limited material and practical assistance. These findings imply that healthcare providers should actively involve both patients and their families in strengthening support systems by offering material, emotional, and informational support, while also promoting better diabetes self-management. In addition, policy interventions should pay particular attention to economically disadvantaged patients by providing targeted medical subsidies and preferential healthcare policies.

The mean health literacy score was 30.47 ± 9.36, which was lower than that reported in previous domestic studies (32), indicating an urgent need to improve health literacy in this population. Among the three dimensions, critical health literacy had the lowest score, which was consistent with previous findings (16). This may be attributable to the relatively low socioeconomic status of rural areas, limited access to medical resources, and generally low educational attainment among rural patients with diabetes, all of which may hinder their ability to obtain, process, and understand health-related information (23). Given these findings, healthcare providers should incorporate health literacy assessment into routine diabetes management in order to identify high-risk individuals and implement targeted interventions (24).

Body weight and BMI were included in the revised description of the study population to provide a more comprehensive characterization of the cohort. The mean body weight and BMI suggested that a considerable proportion of participants were not markedly obese. This is particularly relevant in the East Asian context, where type 2 diabetes may develop even in individuals without severe obesity, partly because of impaired insulin secretory function. Therefore, glycemic control in this population may reflect not only lifestyle-related factors, but also heterogeneity in disease mechanisms, treatment burden, and metabolic vulnerability. Including body weight and BMI helps contextualize the relative contributions of social support, health literacy, and other clinical factors in this rural diabetic population. In addition, family-based emotional, informational, and practical support may play an important role in shaping diabetes management behaviors, especially in settings with limited healthcare access (33).

### Correlation between social support, health literacy and HbA1c control

This study showed a significant negative correlation between social support and HbA1c level. Rural patients with lower levels of social support tended to have higher HbA1c levels. In rural settings, lower educational attainment, more traditional lifestyles, and smaller social networks may increase vulnerability to social isolation (34). These populations may also have more limited access to health information, lower health awareness, and poorer health literacy, all of which may contribute to suboptimal self-management (35, 36). Our findings further showed that both total social support and its subjective and objective dimensions were significantly associated with HbA1c level, suggesting that strengthening social support may enhance self-management capacity and thereby improve glycemic control.

The subscale analysis provided further insight into the relationships between psychosocial factors and glycemic control. Among the social support dimensions, objective and subjective support showed stronger associations with HbA1c than support utilization. This suggests that both the actual availability of support resources and patients’ perceived support may be more closely linked to glycemic control than the mere use of support. Among the health literacy dimensions, communicative health literacy showed the strongest correlation with HbA1c, followed by functional and critical health literacy. This finding suggests that the ability to obtain, understand, and actively use health information in everyday communication may be particularly important for glycemic management in rural patients with diabetes.

This study also demonstrated a significant negative correlation between health literacy and HbA1c level. Patients with better health literacy may be more capable of acquiring disease-related knowledge and developing effective self-management skills, both of which are essential for improved glycemic control (17). By contrast, patients with limited health literacy may be less able to maintain healthy behaviors and make appropriate treatment decisions. Geographic barriers may further aggravate these difficulties. The farther patients live from medical facilities, the greater the challenges they face in accessing health information and maintaining healthy practices. Previous studies have shown that greater distance is associated with fewer follow-up visits and less health guidance from village doctors (25, 37). To address these challenges, rural healthcare providers should strengthen health education, improve access to understandable health information, and develop practical support strategies for vulnerable patients. Mobile health interventions may also help patients increase disease knowledge, correct misconceptions, and strengthen self-management confidence (38).

The present findings are consistent with recent research showing that social support may influence diabetes outcomes through multiple behavioral pathways. A path analytical study among rural individuals with type 2 diabetes in eastern China found that social support was positively associated with self-management and quality of life, both directly and indirectly through self-efficacy (22). Research in Xi’an also reported that social support was associated with medication adherence and glycemic control, suggesting that adherence may be one pathway linking support resources to HbA1c outcomes (11). In addition, mixed-methods evidence from another low-resource setting emphasized the importance of social support for diabetes management beyond purely biomedical factors (12). Together, these findings suggest that social support is not merely a contextual factor, but may also operate through specific self-management and treatment-related pathways.

### Analysis of the mediating effect of health literacy

The mediation analysis showed that health literacy significantly mediated the relationship between social support and glycemic control. In the original model, the indirect effect accounted for 17.49% of the total effect, and in the covariate-adjusted model it remained statistically significant, accounting for 35.05% of the total effect. These findings suggest that the mediating role of health literacy was robust even after adjustment for age, sex, education level, household income, disease duration, number of complications, BMI, and treatment type. Effective glycemic control requires patients to acquire therapeutic knowledge and maintain practical self-management skills over time (39). However, current evidence suggests that diabetes self-management remains suboptimal and often depends on passive education rather than systematic and sustained support, which may hinder glycemic control among rural patients with diabetes.

Although both social support and health literacy were significantly associated with HbA1c level, the explained variance indicates that glycemic control is multifactorial and cannot be fully explained by these two factors alone. In the original model, the explained variance for health literacy and HbA1c was limited, suggesting that additional determinants of glycemic control are likely to exist. In clinical practice, HbA1c may also be influenced by treatment regimen, medication adherence, diabetes-related knowledge, self-management behaviors, diet, physical activity, and access to healthcare services. Therefore, the present model should be interpreted as a partial explanatory framework rather than a comprehensive one.

To address this issue, we further estimated a covariate-adjusted model including age, sex, education level, household income, disease duration, number of complications, BMI, and treatment type. After adjustment, the main mediation pathway remained significant, supporting the robustness of the relationships among social support, health literacy, and HbA1c. Nevertheless, the remaining unexplained variance suggests that additional psychosocial, behavioral, and treatment-related factors should be incorporated in future studies.

In addition to health literacy, other treatment-related factors may also contribute to glycemic control among rural patients with diabetes. For example, patients’ knowledge of their specific treatment regimen, their ability to remember treatment instructions, and their consistency in following prescribed therapy may affect HbA1c independently, although these factors are likely related to health literacy. The relatively broad distribution of HbA1c in the present study (8.60 ± 2.18%) also suggests considerable heterogeneity within the study population. Because treatment-related information, including the use of oral hypoglycemic agents and insulin therapy, was collected in this study, participants may have been receiving different treatment regimens, which could partly explain the variation in glycemic control. Patients receiving oral medication, insulin, or combined therapy may differ in disease severity, treatment burden, self-management demands, and adherence challenges, all of which could influence HbA1c outcomes.

The present mediation findings are consistent with recent literature showing that health literacy influences diabetes outcomes through self-management and behavioral pathways. Recent standards for diabetes self-management education and support have emphasized that person-centered education, psychosocial care, and sustained self-management support are essential for improving diabetes outcomes (20, 40). A recent path analysis study showed that diabetes health literacy was closely linked to self-care and quality of life in patients with type 2 diabetes (14), while a systematic review and meta-analysis indicated that health literacy interventions can improve outcomes in people with diabetes (15). Together, these studies suggest that health literacy is not only associated with HbA1c as an isolated characteristic, but may also operate through a broader pathway involving knowledge acquisition, adherence, self-management behavior, and engagement with healthcare services.

Accordingly, the observed mediation effect of health literacy should be interpreted within a broader clinical context. Social support may influence glycemic control not only by enhancing health literacy, but also by strengthening treatment understanding, promoting adherence, facilitating self-management, and helping patients cope with the complexity of different therapeutic regimens. Recent studies have also highlighted the importance of structural and healthcare-system barriers in diabetes management. A mixed-methods study in Chinese primary care identified barriers at both the provider and patient levels, including resource constraints, service accessibility, and limitations in long-term management support (26). In addition, a recent qualitative study among rural patients with type 2 diabetes found that information barriers, limited disease awareness, and difficulties in accurately perceiving complication risk may hinder timely prevention and self-management (27). These findings reinforce the view that glycemic control in rural populations is influenced not only by individual literacy and support, but also by broader healthcare-system constraints. Future studies should further incorporate treatment-specific knowledge, medication adherence, and regimen complexity to better clarify the mechanisms underlying HbA1c control in rural patients with diabetes (18, 19, 41–43).

### Limitations

This study has several limitations. First, because of its cross-sectional design, causal relationships among social support, health literacy, and HbA1c level cannot be established. Second, although participants were recruited from multiple hospitals, convenience sampling and voluntary participation may have introduced selection bias, which could affect the generalizability of the findings. Third, while social support and health literacy were significantly associated with HbA1c, they did not fully explain glycemic control, suggesting that other important determinants—such as treatment-specific knowledge, medication adherence, dietary management, physical activity, self-management capacity, and healthcare-access factors—were not captured. Fourth, variation in HbA1c results may have occurred because of differences in laboratory equipment across hospitals. Finally, because glycemic control changes over time, future longitudinal studies incorporating a broader range of behavioral and treatment-related variables are needed to better clarify the mechanisms underlying HbA1c control in rural patients with diabetes.

## Conclusion

Social support was significantly associated with HbA1c level, and health literacy played an important mediating role in this relationship. These findings suggest that improving glycemic control in rural patients with diabetes requires not only disease-focused management, but also stronger social support and better health literacy. Accordingly, intervention programs should extend beyond conventional diabetes care to strengthen family ties, friendships, and broader support networks, while helping patients effectively obtain, understand, and apply health information in both offline and online settings. From the perspective of clinical practice and rural health systems, strategies to enhance social support and health literacy should be incorporated into community-based diabetes management programs. At the public health level, targeted and locally appropriate interventions are needed to improve health education, expand access to reliable health information, and support sustainable diabetes management in underserved rural populations. Together, these efforts may help improve glycemic control and reduce diabetes-related health disparities in rural areas.

## Data Availability

The original contributions presented in the study are included in the article/supplementary material. Further inquiries can be directed to the corresponding author.
